# Preditores Clínicos de Insuficiência Cardíaca após IAMCSST: Dados de um País de Renda Média com Acesso Limitado à Intervenção Coronária Percutânea

**DOI:** 10.36660/abc.20240447

**Published:** 2025-03-27

**Authors:** Vinícius C. Fiusa, Andrea D. Stephanus, Victor F. Couto, Gustavo A. Alexim, Thaiene M. M. Severino, Ana Claudia C. Nogueira, Adriana J. B. A. Guimarães, Alexandre Anderson S. M. Soares, Elizabeth Bilevicius, Vivian Batista, Alessandra Staffico, Andrei C. Sposito, Luiz Sérgio F. de Carvalho

**Affiliations:** 1 Universidade Católica de Brasília Brasília DF Brasil Universidade Católica de Brasília, Brasília, DF – Brasil; 2 Escola Superior de Ciências da Saúde Brasília DF Brasil Escola Superior de Ciências da Saúde, Brasília, DF – Brasil; 3 Instituto Aramari Apo Brasília DF Brasil Instituto Aramari Apo, Brasília, DF – Brasil; 4 Viatris Brasil São Paulo SP Brasil Viatris Brasil, São Paulo, SP – Brasil; 5 Creighton University Pharmacy Sciences Omaha Nebraska EUA Creighton University - Pharmacy Sciences, Omaha, Nebraska – EUA; 6 Universidade Estadual de Campinas Campinas SP Brasil Universidade Estadual de Campinas,Campinas, SP – Brasil; 7 Clarity Healthcare Intelligence Jundiaí SP Brasil Clarity Healthcare Intelligence, Jundiaí, SP – Brasil

**Keywords:** Insuficiência Cardíaca, Infarto do Miocárdio com Supradesnível do Segmento ST, Fatores de Risco, Trombólise Farmacológica

## Abstract

**Fundamento:**

A insuficiência cardíaca (IC) é uma complicação comum do infarto do miocárdio com supradesnível do segmento ST (IAMCSST) em países de rendas baixa e média, nos quais a mortalidade por doença cardiovascular é desproporcionalmente alta. A intervenção coronária percutânea primária (ICP) reduziu a incidência de IC pós-IAMCSST em países desenvolvidos. Entretanto, em países de rendas baixa e média, o acesso a essa abordagem é baixo, e dados desses locais são escassos.

**Objetivos:**

Identificar preditores de IC após IAMCSST em um país de renda baixa/média com acesso limitado à ICP, visando melhores manejo e desfechos.

**Métodos:**

Este estudo retrospectivo do tipo coorte analisou 2467 pacientes com IAMCSST admitidos em dois hospitais públicos brasileiros entre janeiro de 2015 e fevereiro de 2020. Todos os participantes receberam trombólise farmacológica e foram submetidos à angiografia coronária dentro de 48 horas após a admissão. O desfecho primário foi IC sintomática, definida como dispneia com evidência de congestão do raio-X de tórax, entre 48 horas de admissão até a alta. Regressão logística binária stepwise foi usada para identificar preditores de IC. A significância estatística foi definida como p-valores < 0,05.

**Resultados:**

A idade média da população foi de 58,3±12,6 anos, 61,9% eram do sexo masculino, e 39,9% desenvolveram IC após IAMCSST. A IC foi mais frequente nos homens com idade mais avançada e com doença cardiovascular-renal-metabólica, infartos maiores, e envolvimento da artéria descendente anterior esquerda. Geralmente, os pacientes não recebiam prescrição adequada dos medicamentos na alta, principalmente de antagonistas de aldosterona (11,0%). A IC foi notavelmente mais frequente entre os indivíduos com falha no tratamento de trombólise (47,0%).

**Conclusões:**

Esta coorte regionalmente-representativa de um país de renda baixa/média, com acesso limitado à ICP, mostrou que homens mais velhos com doença cardiovascular-renal-metabólica são particularmente vulneráveis à IC pós-IAMCSST, e que a farmacoterapia para IC na alta necessita ser otimizada. A alta incidência de IC entre os pacientes com falha de trombólise destaca a necessidade de expandir a disponibilidade da IPC.

## Introdução

O infarto do miocárdio com supradesnível do segmento ST (IAMCSST) continua sendo um grande fator contribuinte para morbidade e mortalidade no mundo.^1,2^ O IAMCSST geralmente leva à insuficiência cardíaca (IC), com altos custos em saúde e um grande impacto sobre a qualidade de vida e a produtividade dos pacientes.^3,4^

Nas últimas décadas, avanços no cuidado cardiovascular agudo e nas técnicas de revascularização, particularmente a intervenção coronária percutânea primária (ICPP), levaram a melhores desfechos em países de alta renda, tais como Estados Unidos, Suécia, Dinamarca e Austrália.^5-10^ Contudo, esta estratégia padrão-ouro geralmente não está disponível em países de rendas baixa e média, e pacientes nesses locais recebem principalmente trombólise farmacológica, ou mesmo nenhuma terapia de reperfusão.

Artigos recentes exploraram fatores de risco para IC pós-IAMCSST após o advento da ICPP.^11-13^ Estudos da era trombolítica também investigaram esses tópicos em países de alta renda.^2^ No entanto, em nosso conhecimento, não existe literatura contemporânea sobre IC após IAMCSST em países de rendas baixa e média, com acesso limitado ou nenhum acesso à ICP.

Portanto, este grande estudo multicêntrico tem como objetivo identificar e quantificar preditores de IC após IAMCSST em um típico país de renda baixa/média com acesso limitado à ICP. Identificar esses preditores abre caminho para estratégias mais eficazes e melhores desfechos nesses ambientes de recursos escassos.

## Métodos

### Delineamento do estudo, população e ética

**Figura 1:**
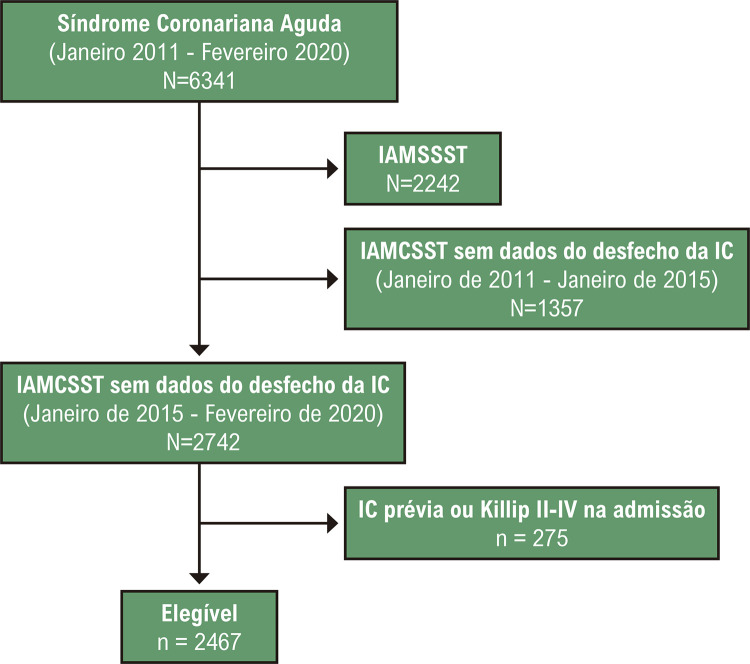
– Fluxograma STROBE; IAMCSST: infarto agudo do miocárdio com supradesnível do segmento ST; IM: infarto do miocárdio; IAMSSST: infarto agudo do miocárdio sem supradesnível do segmento ST; IC: insuficiência cardíaca.

O presente estudo utilizou dados do Registro Cardiovascular de Brasília para Qualidade da Assistência e Resultados (B-CaRe:QCO, *Brasilia Cardiovascular Registry for Quality of Care and Outcomes*), um banco de dados retrospectivo de 6341 pacientes admitidos por síndrome coronariana aguda em dois hospitais públicos terciários do Distrito Federal entre janeiro de 2011 e fevereiro de 2020.^14,15^ Incluímos somente o subgrupo de pacientes internados por IAMCSST, e somente aqueles internados após janeiro de 2015, uma vez que os dados sobre IC incidente não estavam disponíveis para pacientes internados anteriormente (n=2722). Também excluímos 275 pacientes com IC no basal e pacientes com classe II-IV de Killip na admissão hospitalar. A coorte final foi composta de 2467 pacientes elegíveis, conforme descrito no fluxograma STROBE (Figura 1).

Todos os participantes receberam trombólise farmacológica e foram submetidos à coronariografia dentro de 48 horas da admissão, ou no Hospital de Base do Distrito Federal (HBDF) ou no Instituto de Cardiologia e Transplantes do Distrito Federal (ICTDF), ambos em Brasília, Brasil. Entre 2015 e 2019, essas duas instituições realizaram cerca de 99% de todas as angiografias coronárias nos pacientes com IAMCSST no sistema de saúde pública do Distrito Federal.

A metodologia de pesquisa seguiu a Declaração de Helsinki da *World Medical Association* (WMA), e foi aprovada pelo comitê de ética do Instituto de Gestão Estratégica de Saúde do Distrito Federal (IGESDF), que permitiu ausência de termo de consentimento dada à coleta de dados desidentificados (número de aprovação 28530919.0.1001.8153).

### Coleta de dados e desfecho primário

Os dados foram coletados por meio de análise de prontuários médicos digitais utilizando formulários padronizados. As categorias dos dados incluíram: dados demográficos, história clínica, características do IAMCSST índice, resultados laboratoriais, detalhes do tratamento, e desfechos da internação. Os exames laboratoriais foram conduzidos seguindo procedimentos clínicos padrões. A gravidade da anatomia coronária, a extensão da doença aterosclerótica, tratamentos angiográficos, grau de fluxo TIMI, e grau de *blush* miocárdico foram determinados por análise de relatórios médicos.

O desfecho primário foi IC incidente durante a internação, definida como dispneia com evidência de congestão na radiografia de tórax entre 48 horas após a admissão e a alta.

### Análise estatística

As características da população foram comparadas entre os grupos com e sem IC. As variáveis categóricas foram representadas em contagens e porcentagens, e comparadas usando o teste do qui-quadrado de Pearson (ou o teste exato de Fisher para variáveis com menos de 10 ocorrências). As variáveis contínuas foram representadas como média e desvio padrão, e comparadas usando o teste t de Student independente.

A normalidade foi testada usando o teste de Kolmogorov-Smirnov e confirmada por análise do gráfico. As variáveis também foram testadas quanto à ausência de multicolinearidade. A regressão logística binária (razão de verossimilhança) foi usada para identificar e quantificar preditores de IC sintomática pós-IAMCSST durante a internação. A razão de chances (*odds ratio*, OR) e respectivo Intervalo de Confiança de 95% (IC95%) foram calculados para cada preditor identificado. O teste de Hosmer-Lemeshow foi usado para verificar se o modelo se ajusta bem aos dados.

Significância estatística foi definida por p-valores < 0,05. O programa Microsoft Excel 2021 foi usado para manejo dos dados. As análises foram realizadas usando o IBM SPSS 26 para Windows.

## Resultados

Entre janeiro de 2015 e fevereiro de 2020, 2467 indivíduos foram admitidos por IAMCSST em dois hospitais públicos terciários no Distrito Federal. A idade média dos pacientes foi 58,3±12,6 anos, e a maioria (61,9%, n=1520) dos pacientes era do sexo masculino (n=1520). A IC pós-IAMCSST foi observada em 984 pacientes (39,9%).

### Características basais da população

A IC foi mais frequente nos pacientes do sexo masculino e mais velhos (Tabela 1). A probabilidade de desenvolver essa condição foi significativamente mais baixa nos pacientes com idade inferior a 60 anos (OR: 0,581 IC95% 0,491-0,687) em comparação àqueles com idade entre 60 e 79 anos (OR: 2,241, IC95% 1,541-3,259). A obesidade foi mais prevalente no grupo com IC, assim como hipertensão, diabetes, hipotiroidismo, doença renal crônica, doença arterial coronariana (DAC) prévia e ICP prévia.

**Tabela 1:** – Características basais

Variable	IC incidente após IAMCSST		Valor p	OR (IC95%)
	Não (n=1483)	Sim (n=984)		
**Dados demográficos**				
Sexo masculino, n(%)	885(59,6)	635(64,5)	0,015*	1,229(1,040-1,452)
Idade (anos), μ±SD	57,5±13,3	59,5±11,4	<0,001†	-
Idade <60 anos, n(%)	1,026(69,2)	557(56,6)	<0,001*	0,581(0,491-0,687)
Idade 60-79 anos, n(%)	408(27,5)	357(36,3)	<0,001*	1,500(1,262-1,783)
Idade ≥80 anos, n(%)	49(3,3)	70(7,1%)	<0,001*	2,241(1,541-3,259)
ICM (kg/m^2^), μ±SD	26,73±4,50	27,13±4,57	0,032†	-
Baixo peso (<18,5kg/m^2^), n(%)	22(1,5)	7(0,7)	0,088‡	0,475(0,202-1,115)
Peso normal (18,5-24,9kg/m^2^), n(%)	559(37,8)	347(35,3)	0,204*	0,897(0,758-1,061)
Sobrepeso (25-29,9kg/m^2^), n(%)	613(41,5)	403(41,0)	0,814*	0,980(0,832-1,155)
Obesidade I (30-34,9kg/m^2^), n(%)	219(14,8)	166(16,9)	0,166*	1,168(0,937-1,456)
Obesidade II (35-39,9kg/m^2^), n(%)	45(3,0)	46(4,7)	0,035*	1,563(1,028-2,377)
Obesidade III (≥40kg/m^2^), n(%)	20(1,4)	14(1,4)	0,882*	1,053(0,529-2,095)
**História clínica**				
Obesidade, n(%)	260(17,5)	219(22,3)	0,004*	1,347(1,101-1,647)
Hipertensão, n(%)	863(58,2)	614(62,4)	0,037*	1,192(1,011-1,406)
Diabetes, n(%)	387(26,1)	349(35,5)	<0,001*	1,557(1,307-1,853)
Dislipidemia, n(%)	709(47,8)	507(51,5)	0,071*	1,160(0,988-1,363)
História de tabagismo, n(%)	960(64,7)	610(62,0)	0,166*	0,889(0,752-1,050)
História de elitismo, n(%)	183(12,3)	118(12,0)	0,796*	0,968(0,756-1,239)
História de uso de drogas ilícitas, n(%)	64(4,3)	31(3,2)	0,141*	0,721(0,466-1,116)
História familiar de DCV, n(%)	319(21,5)	197(20,0)	0,373*	0,913(0,748-1,115)
Hipotiroidismo, n(%)	73(4,9)	72(7,3)	0,013*	1,525(1,090-2,134)
**Lesão de órgão alvo**				
DRC, n(%)	68(4,6)	90(9,1)	<0,001*	2,095(1,512-2,902)
DAC prévia, (%)	399(26,9)	324(32,9)	0,001*	1,334(1,119-1,590)
Angina prévia, n(%)	330(22,3)	243(24,7)	0,159*	1,146(0,948-1,385)
IC prévio, n(%)	105(7,1)	128(13,0)	<0,001*	1,962(1,495-2,575)
ICP prévia, n(%)	47(3,2)	70(7,1)	<0,001*	2,340(1,602-3,418)
CABG prévio, n(%)	26(1,8)	17(1,7)	0,962*	0,985(0,532-1,825)
AVC prévio, n(%)	51(3,4)	44(4,5)	0,192*	1,314(0,871-1,984)
DAP prévia, n(%)	57(3,8)	51(5,2)	0,111*	1,368(0,929-2,013)

*Teste do qui-quadrado de Pearson. †Teste t de Student para amostras independentes. ‡ Teste exato de Fisher; valores p em negrito indicam significância estatística (p<0,05); OR: Odds Ratio; IC: Intervalo de Confiança; n: número de indivíduos. μ: valor médio; DP: desvio padrão; IC: insuficiência cardíaca; IAMCSST: Infarto Agudo do Miocárdio com Supradesnível do Segmento ST; IMC: índice de massa corporal; DCV: doença cardiovascular; DRC: doença renal crônica; DAC: doença arterial coronariana; ICP: intervenção coronária percutânea; CABG: bypass da artéria coronária; AVC: acidente vascular cerebral; DAP: doença arterial periférica.

### Características do IAM índice

Em relação á sintomatologia na admissão, os pacientes que desenvolveram IC apresentaram-se com síncope e parada cardíaca com mais frequência (Tabela 2). Esses pacientes também apresentaram valores mais baixos de pressão arterial sistólica (PAS) e diastólica, bem como escores GRACE e CRUSADE mais altos. Ainda, os pacientes com IC apresentaram, com maior frequência, infarto do miocárdio anterior e bloqueio de ramo direito no eletrocardiograma (ECG) na admissão. Ondas Q patológicas foram mais comuns nas derivações V1-V4 (anterior), V1-V6 (anterolateral), e V4R (direita), e menos comum nas derivações V5-V6 (lateral) e DII-DIII/AVF (inferior).

**Tabela 2:** – Características do Infarto Agudo do Miocárdio com supradesnível do segmento ST (IAMCSST) índice

Variável	IC incidente após IAMCSST		Valor p	OR (IC95%)
	Não (n=1483)	Sim (n=984)		
**Sintomatologia na admissão**				
Síncope, n (%)	12(0,8)	20(2,0)	0,009*	2,543(1,238-5,226)
Parada cardíaca, n (%)	4(0,3)	22(2,2)	<0,001‡	8,450(2,903-24,597)
PAS (mmHg), μ±DP	136,3±25,1	129,7±30,0	<0,001†	-
PAD (mmHg), μ±DP	83,1±16,3	81,1±20,5	<0,001†	-
**Estratificação de risco na admissão**				
Escore GRACE, μ±DP	99,2±24,5	141,9±43,1	<0,001†	-
Escore CRUSADE, μ±DP	21,6±12,2	33,7±15,0	<0,001†	-
**ECG na admissão**				
IM na parede anterior, n (%)	495(33,4)	628(63,8)	<0,001*	3,521(2,974-4,169)
Bloqueio de ramo direito, n (%)	6(0,4)	16(1,6)	0,002‡	4,069(1,587-10,435)
Bloqueio de ramo esquerdo, n (%)	4(0,3)	6(0,6)	0,211‡	2,268(0,638-8,059)
Bloqueio AV de 2º/3º grau, n (%)	5(0,3)	7(0,7)	0,240‡	2,118(0,670-6,692)
Via septal (V1-V3), ondas Q, n (%)	80(5,4)	55(5,6)	0,835*	1,038(0,729-1,478)
Via anterior (V1-V4), ondas Q, n (%)	191(12,9)	191(19,4)	<0,001*	1,629(1,309-2,029)
Via anterolateral (V1-V6), ondas Q, n (%)	225(15,2)	383(38,9)	<0,001*	3,563(2,943-4,314)
Via lateral (V5-V6), ondas Q, n (%)	80(5,4)	30(3,0)	0,006*	0,551(0,360-0,846)
Via direita (V4R), ondas Q, n (%)	129(8,7)	110(11,2)	0,041*	1,321(1,010-1,727)
Via inferior (DI, DII, AVF), ondas Q, n (%)	649(43,8)	211(21,4)	<0,001*	0,351(0,292-0,421)
**Resultados laboratoriais**				
cTn-US na admissão (ng/L), μ±DP	5,042±5,978	8,436±9,071	<0,001†	-
Pico cTn-US (ng/L), μ± DP	6,164±5,658	11,145±10,992	<0,001†	-
AST (U/L), μ± DP	167±139	267±198	<0,001†	-
ALT (U/L), μ± DP	49,4±48,5	95,7±185,7	<0,001†	-
Creatinina (mg/dL), μ±DP	0,92±0,40	1,11±0,87	<0,001†	-
TFG (mL/min), μ±DP	96,5±34,3	85,4±35,3	<0,001†	-
Glicemia (mg/dL), μ±DP	132,6±56,5	166,0±85,6	<0,001†	-
HbA1c (%), μ±DP	6,55±1,81	6,95±2,23	<0,001†	-
TSH (mIU/L), μ±DP	2,16±3,99	2,81±6,21	0,007†	-
FT4 (ng/dL), μ±DP	1,29±0,63	1,30±0,62	0,744†	-
Colesterol total (mg/dL), μ±DP	201,49±45,5	198,2±48,5	0,111†	-
HDL-C (mg/dL), μ±DP	41,5±12,3	41,3±12,5	0,637†	-
LDL-C (mg/dL), μ±DP	130,0±39,5	125,2±40,9	0,010†	-
Triglicerídeos (mg/dL), μ±DP	158,2±109,7	158,9±119,2	0,876†	-
Hemoglobina (g/dL), μ±DP	14,55±1,70	14,50±1,96	0,053†	-
Hematócrito (%), μ±DP	43,03±4,84	42,86±5,72	0,448†	-
TP (s), μ±DP	85,3±12,2	81,4±15,4	<0,001†	-
TTP (s), μ±DP	34,9±13,0	38,0±20,2	<0,001†	-

*Teste do qui-quadrado de Pearson. †Teste t de Student para amostras independentes. ‡ Teste exato de Fisher; valores p em negrito indicam significância estatística (p<0,05); OR: Odds Ratio; IC: Intervalo de Confiança; n: número de indivíduos. μ: valor médio; DP: desvio padrão; IC: insuficiência cardíaca; IAMCSST: Infarto Agudo do Miocárdio com Supradesnível do Segmento ST; PAS: pressão arterial sistólica; PAS: pressão arterial diastólica; IM: infarto do miocárdio; cTn-US: Troponina cardíaca ultrassensível; AV: atrioventricular; AST: aspartato aminotransferase; ALT: alanina aminotransferase; TFG: taxa de filtração glomerular, HbA1c: hemoglobina A1c; TSH: hormônio tireoestimulante; FT4: tiroxina livre; HDL-C: lipoproteína de alta densidade; LDL-C: lipoproteína de baixa densidade; TP: tempo de protrombina;. TTP: tempo de tromboplastina parcial.

A troponina cardíaca ultrassensível (cTn-US) na admissão foi mais alta no grupo com IC, assim como seus valores de pico e níveis de aminotransferase (AST), indicando lesão miocárdica mais extensa. A disfunção renal também foi mais comum no grupo com IC, com valores mais altos de creatinina e Taxa de Filtração Glomerular (TFG). Os pacientes com IC também apresentaram níveis elevados de glicose e hemoglobina A1c (HbA1c), e níveis mais baixos de lipoproteína de baixa densidade (LDL). Ainda, um Tempo de Protrombina (TP) mais curto e um Tempo de Tromboplastina Parcial (TTP) prolongado foram observados entre os pacientes com IC.

### Detalhes do tratamento

A estratégia de tratamento fármaco-invasiva foi fortemente e inversamente associada com IC pós-IAMCSST incidente (OR: 0,280; IC95%: 0,235-0,334) (Tabela 3). Em contraste, indivíduos submetidos à ICP de resgate, após não terem preenchido os critérios de perfusão, desenvolveram IC com mais frequência (OR: 3,920; IC95%: 3,269-4,699). Tempos mais curtos entre o início dos sintomas e a ida ao hospital e entre trombólise e o cateterismo foram vistos no grupo com IC, provavelmente refletindo a priorização de apresentações mais graves, dada a capacidade limitada de nosso sistema público de saúde.

**Tabela 3:** – Detalhes do tratamento

Variável	IC incidente após IAMCSST		Valor p	OR (IC95%)
	Não (n=1483)	Não (n=1483)		
**Detalhes do tratamento**				
Fármaco-invasiva, n (%)	1,164(78,5)	498(50,6)	<0,001*	0,280(0,235-0,334)
ICP de resgate, n (%)	273(18,4)	462(47,0)	<0,001*	3,920(3,269-4,699)
2º cateterismo, n (%)	110(7,5)	91(9,3)	0,106*	1,270(0,950-1,698)
Angioplastia no 2º cateterismo, n (%)	81(7,6)	61(8,1)	0,669*	1,078(0,763-1,524)
Início de sintoma -internação (min), μ±DP	158±135	146±126	0,036†	-
Porta-agulha (min), μ±DP	181±3,436	98±92	0,449†	-
Trombólise -cateterismo (min), μ±DP	1,392±1,220	1,059±1,186	<0,001†	-
**Detalhes do cateterismo e ICP**				
Uso de inibidor de GPIIb/IIIa, n (%)	50(3,4)	66(6,7)	<0,001*	2,061(1,414-3,003)
Uso de nitroprussiato, n (%)	5(0,3)	10(1,0)	0,034‡	3,035(1,034-8,906)
Uso de adenosina, n (%)	38(2,6)	61(6,2)	<0,001*	2,513(1,662-3,800)
Número de stents convencionais, μ±DP	0,83±0,73	0,93±0,75	0,001†	-
Número de stents farmacológicos, μ±DP	0,09±0,35	0,07±0,34	0,136†	-
Fluxo TIMI-3 após ICP, n (%)	1,058(83,4)	601(69,0)	<0,001*	0,444(0,361-0,545)
GBM 3 após ICP, n (%)	845(69,7)	393(49,0)	<0,001*	0,418(0,348-0,503)
Duração do cateterismo (min), μ±DP	90±1,181	62±29	0,460†	-
**Ecocardiografia 48-72h após admissão**				
FEVE (%), μ±SD	54,9±7,4	41,0±9,8	<0,001†	-
FEVE<40%, n (%)	1(0,1)	367(42,7)	<0,001‡	851,8(119,3-6,080,8)
Hipocinesia anterior, n (%)	192(12,9)	254(25,8)	<0,001*	2,340(1,900-2,881)
Hipocinesia septal, n (%)	373(25,2)	257(26,1)	0,590*	1,052(0,875-1,265)
Hipocinesia lateral, n (%)	125(8,4)	160(16,3)	<0,001*	2,110(1,644-2,707)
Hipocinesia direita, n (%)	3(0,2)	15(1,5)	<0,001‡	7,637(2,205-26,449)
Hipocinesia inferior, n (%)	396(26,7)	211(21,4)	0,003*	0,749(0,619-0,907)
Hipocinesia apical, n (%)	77(5,2)	91(9,2)	<0,001*	1,861(1,358-2,549)
Hipocinesia dorsal, n (%)	273(18,4)	130(13,2)	0,001*	0,675(0,538-0,846)
Acinesia anterior, n (%)	124(8,4)	373(37,9)	<0,001*	6,691(5,345-8,374)
Acinesia septal, n (%)	251(16,9)	467(47,5)	<0,001*	4,434(3,686-5,333)
Acinesia lateral, n (%)	39(2,6)	90(8,1)	<0,001*	3,277(2,215-4,848)
Acinesia direita, n (%)	0(0,0)	2(0,2)	0,159‡	0,398(0,380-0,418)
Acinesia inferior, n (%)	382(25,8)	264(26,8)	0,554*	1,057(0,880-1,269)
Acinesia apical, n (%)	188(12,7)	431(43,8)	<0,001*	5,369(4,404-6,545)
Acinesia dorsal, n (%)	108(7,3)	105(10,7)	0,003*	1,521(1,148-2,016)
**Farmacoterapia na alta (ou óbito)**				
Inibidores de ECA, n (%)	661(44,6)	413(41,9)	0,202*	0,899(0,764-1,058)
Bloqueadores de receptor de angiotensina, n (%)	351(23,7)	167(16,9)	<0,001*	0,659(0,537-0,809)
Betabloqueadores, n (%)	1,003(67,6)	656(66,6)	0,616*	0,957(0,806-1,136)
Antagonistas de aldosterona, n (%)	64(4,3)	109(11,0)	<0,001*	2,762(2,005-3,803)
Furosemida, n (%)	86(5,8)	110(11,1)	<0,001*	2,044(1,522-2,744)
Bloqueadores de canais de cálcio, n (%)	320(21,6)	145(14,7)	<0,001*	0,628(0,506-0,779)
Estatinas, n (%)	1,226(82,7)	815(82,8)	0,920*	1,010(0,816-1,251)
Ácido acetilsalicílico, n (%)	1,449(97,7)	965(98,8)	0,543*	1,191(0,675-2,101)
Clopidogrel, n (%)	1,035(69,8)	667(67,7)	0,291*	0,910(0,765-1,083)
Prasugrel, n (%)	214(14,4)	260(26,4)	<0,001*	2,129(1,738-2,607)
Ticagrelor, n (%)	33(2,2)	35(3,5)	0,047*	1,620(1,000-2,625)
Anticoagulantes, n (%)	46(3,1)	56(5,6)	0,001*	1,885(1,265-2,808)

*Teste do qui-quadrado de Pearson. †Teste t de Student para amostras independentes. ‡ Teste exato de Fisher; valores p em negrito indicam significância estatística (p<0,05); OR: Odds Ratio; IC: intervalo de confiança; n: número de indivíduos. μ: valor médio; DP: desvio padrão; IC: insuficiência cardíaca; IAMCSST: infarto agudo do miocárdio com supradesnível do segmento ST; ICP: intervenção coronária percutânea; FEVE: fração de ejeção ventricular esquerda; ECA: enzima conversora de angiotensina; GBM: grau de blush miocárdico.

Quanto aos detalhes da ICP, IC incidente foi mais comum entre os pacientes que receberam inibidores de GPIIb/IIIa e nitroprussiato durante o cateterismo, e naqueles com mais *stents* convencionais implantados. Um fluxo TIMI 3 final e *blush* miocárdico grau 3 foram menos frequentemente alcançados no grupo com IC.

Os pacientes com IC apresentaram uma média mais baixa de fração de ejeção ventricular esquerda (FEVE) e fração de ejeção reduzida (FEr) (FEVE <40%) no ecocardiograma realizado 48-72 horas após admissão. Mudanças na motilidade no grupo com IC foram associados principalmente a envolvimento da artéria descendente anterior esquerda (ADAE).

Na alta hospitalar (ou morte), os pacientes com IC receberam menos prescrição de bloqueadores do recetor da angiotensina II (BRAs) e bloqueadores de canais de cálcio. Por outro lado, esse grupo recebeu, com maior frequência, antagonistas de aldosterona, furosemida, prasugrel, ticagrelor e anticoagulantes.

### Desfechos hospitalares

Angina e IM recorrentes ocorreram com maior frequência no grupo com IC (Tabela 4). Outras complicações cardiovasculares também foram associadas com IC incidente, incluindo: fibrilação atrial, bloqueio atrioventricular total, regurgitação mitral, parada cardíaca, acidente vascular cerebral isquêmico e hemorrágico, sangramento menor e maior, e transfusão de sangue. A média de tempo de internação foi mais longa no grupo com IC, além de uma maior ocorrência de mortalidade hospitalar por todas as causas.

**Tabela 4:** – Desfechos na internação

Variável	IC incidente após IAMCSST		Valor p	OR (IC95%)
	Não (n=1483)	Sim (n=984)		
Angina recorrente, n (%)	32(2,2)	35(3,6)	0,036*	1,672(1,028-2,720)
IM recorrente, n (%)	12(0,8)	28(2,8)	<0,001*	3,590(1,817-7,095)
Atrial fibrillation, n (%)	31(2,1)	55(5,6)	<0,001*	2,773(1,772-4,339)
Bloqueio atrioventricular de 3º grau, n (%)	48(3,2)	70(7,1)	<0,001*	2,290(1,571-3,337)
Regurgitação mitral, n(%)	3(0,2)	9(0,9)	0,017‡	4,554(1,230-16,863)
Parada cardíaca, n(%)	5(0,3)	24(2,4)	<0,001‡	7,385(2,808-19,422)
AVC isquêmico, n(%)	6(0,4)	21(2,1)	<0,001‡	5,368(2,159-13,348)
AVC hemorrágico, n(%)	3(0,2)	13(1,3)	0,001‡	6,605(1,877-23,238)
Sangramento menor, n(%)	28(1,9)	38(3,9)	0,003*	2,087(1,272-3,424)
Sangramento maior, n(%)	11(0,7)	51(5,2)	<0,001*	7,315(3,793-14,106)
Transfusão sanguínea, n(%)	7(0,5)	31(3,2)	<0,001‡	6,859(3,008-15,639)
Tempo de internação (dias), μ±DP	4,01±3,15	6,59±7,36	<0,001†	-
Morte hospitalar por todas as causas, n(%)	12(0,8)	110(11,2)	<0,001*	15,428(8,453-28,160)

*Teste do qui-quadrado de Pearson. †Teste t de Student para amostras independentes. ‡ Teste exato de Fisher; valores p em negrito indicam significância estatística (p<0,05); OR: Odds Ratio; IC: Intervalo de Confiança; n: número de indivíduos. μ: valor médio; DP: Desvio Padrão; IC: Insuficiência Cardíaca; IAMCSST: Infarto Agudo do Miocárdio com Supradesnível do Segmento ST; AVC: Acidente Vascular Cerebral

### Predição de IC pós-IAMCSST durante a internação

Conduzimos uma regressão logística binária (razão de verossimilhança) para identificar e quantificar preditores significativos de IC incidente durante a internação após o IAMCSST (Tabela 5). Essa análise resultou em um modelo estatisticamente significativo [Nagelkerke R^2^=0,550; p=0,039] que classificou corretamente 82,3% dos casos. O teste de Hosmer-Lemeshow indicou uma boa adequação do modelo (p=0,746).

**Tabela 5:** – Preditores de Insuficiência Cardíaca (IC) após Infarto Agudo do Miocárdio com Supradesnível do Segmento ST (IAMCSST) durante internação identificados por regressão logística binária stepwise (razão de verossimilhança)

Variável	B	EP	Wald	Valor p	OR (IC95%)
**Preditores de risco para IC após IAMCSST durante internação**					
AST (incrementos de 100U/L)	0,373	0,061	37,398	<0,001*	1,453(1,289-1,637)
Via direita (V4R); ondas Q (no ECG de admissão)	1,770	0,318	30,920	<0,001*	5,868(3,145-10,950)
Acinesia anterior (48-72h após admissão)	1,159	0,304	14,549	<0,001*	3,187(1,757-5,782)
ICP de resgate	0,787	0,215	13,400	<0,001*	2,197(1,441-3,348)
Hipocinesia apical (48-72h após admissão)	1,260	0,349	13,005	<0,001*	3,524(1,777-6,987)
Acinesia septal (48-72h após admissão)	0,869	0,249	12,207	<0,001*	2,384(1,464-3,880)
Duração do cateterismo (h)	0,750	0,216	12,052	0,001*	2,117(1,386-3,233)
Hipotireoidismo	1,114	0,361	9,547	0,002*	3,047(1,503-6,177)
IM prévio	0,915	0,307	8,875	0,003*	2,497(1,368-4,559)
Acinesia dorsal (48-72h após admissão)	0,940	0,329	8,157	0,004*	2,560(1,343-4,880)
Glicemia (incrementos de 100mg/dL)	0,371	0,147	6,351	0,012*	1,449(1,086-1,933)
Bloqueio de ramo direito (no ECG na admissão)	2,442	1,022	5,712	0,017*	11,501(1,552-85,245)
Hipocinesia lateral (48h após admissão)	0,705	0,297	5,621	0,018*	2,023(1,130-3,622)
Creatinina (mg/dL)	0,566	0,249	5,168	0,023*	1,761(1,081-2,870)
História de tabagismo	0,478	0,211	5,142	0,023*	1,613(1,067-2,438)
Obesidade	0,492	0,236	4,343	0,037*	1,636(1,030-2,600)
IM na parede anterior (ECG na admissão)	0,553	0,267	4,286	0,038*	1,739(1,030-2,936)
Acinesia apical (48-72h após admissão)	0,544	0,286	3,609	0,057*	1,722(0,983-3,017)
**Preditores de proteção para IC após IAMCSST durante internação**					
PAS (mmHg) (na admissão)	-0,017	0,004	21,893	<0,001*	0,983(0,976-0,990)
Idade <60 anos	-0,868	0,204	18,131	<0,001*	0,420(0,282-0,626)
Fluxo TIMI-3 após ICP	-0,880	0,285	9,522	0,002*	0,415(0,237-0,726)
TP (s)	-0,018	0,007	7,161	0,007*	0,982(0,969-0,995)

* Regressão logística binária stepwise (razão de verossimilhança); valores p em negrito indicam significância estatística (p<0,05); EP: erro padrão; OR: Odds Ratio; IC: intervalo de confiança; IC: insuficiência cardíaca; IAMCSST: infarto agudo do miocárdio com supradesnível do segmento ST; IM: infarto do miocárdio; cTn-US: troponina cardíaca ultrassensível; AST: aspartato aminotransferase; TP: tempo de protrombina; PAS: pressão arterial sistólica; ICP: intervenção coronária percutânea; ECG: eletrocardiograma.

Preditores de risco de IC incidente após IAMCSST incluem: AST (em aumentos de 100U/L), ondas Q patológicas em V4R, acinesia anterior, ICP de resgate, hipocinesia apical, acinesia apical, duração do cateterismo (em horas), hipotiroidismo, IM prévio, acinesia dorsal, glicemia (em aumentos de 100 mg/dL), bloqueio de ramo esquerdo, hipocinesia lateral, creatinina (mg/dL), história de tabagismo, obesidade, IM prévio, e acinesia apical.

Quanto aos preditores de proteção, os seguintes fatores foram identificados: PAS mais alta na admissão, idade inferior a 60 anos, fluxo TIMI-3 após ICP, e TP (em segundos).

## Discussão

Em nosso conhecimento, este é um estudo pioneiro em identificar preditores de risco e de proteção para IC pós-IAMCSST em um país de renda baixa/média com acesso limitado à ICPP (Figura Central). Os pacientes que desenvolveram IC apresentaram um claro perfil de risco cardiovascular aumentado, marcado por idade mais avançada, sexo masculino, disfunção metabólica, e lesão de órgão alvo estabelecida. Também se observou que infartos maiores e envolvimento de ADAE foram preditores de IC. Além disso, medicamentos que melhoram o remodelamento cardíaco, particularmente antagonistas de aldosterona, foram nitidamente subprescritos a pacientes com IC. Finalmente, a elevada incidência de IC, principalmente entre os pacientes em que a trombólise não foi efetiva, destaca a necessidade de se expandir a viabilidade da ICPP em países de renda baixa/média como o Brasil

**Figura Central:**
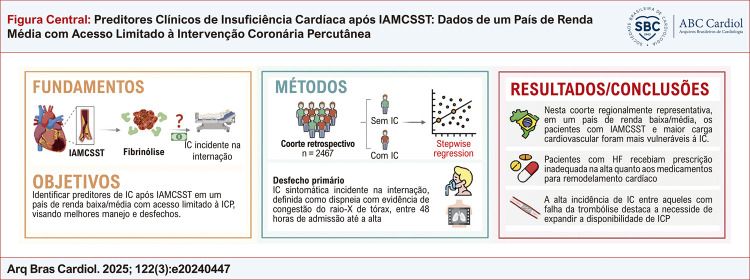
Preditores Clínicos de Insuficiência Cardíaca após IAMCSST: Dados de um País de Renda Média com Acesso Limitado à Intervenção Coronária Percutânea

Resumo do delineamento e dos resultados do estudo. IAMCSST: infarto do miocárdio com supradesnível do segmento ST; IC: insuficiência cardíaca; ICP: intervenção coronária percutânea.

A observação de um risco cardiovascular-renal-metabólico aumentado nos pacientes que desenvolveram IC após o IAMCSST está de acordo com estudos prévios.^13,16-18^ Um dado interessante foi que todos os componentes cardiovasculares (hipertensão, DAC prévia), renais (doença renal crônica), e metabólicos (obesidade, diabetes, hipotiroidismo) foram mais prevalentes no grupo com IC. A exceção foi dislipidemia, em que os paciente com IC apresentaram níveis mais baixos de LDL. Esse resultado foi observado em outros estudos, e é principalmente explicado pela resposta inflamatória associada ao infarto.^13,18,19^

Nossos achados também corroboram as associações bem estabelecidas do tamanho do infarto e do IM anterior com IC após IM.^13,18,20,21^ Na admissão hospitalar, uma pressão arterial mais baixa sugere lesão miocárdica grave o suficiente para causar algum grau de disfunção hemodinâmica. Níveis séricos mais altos de cTn-US e AST, que se correlacionam com uma área infartada maior, também foram observados no grupo com IC.^22-25^ O envolvimento da ADAE exerceu um importante papel em nosso modelo preditivo, abrangendo vários componentes, tais como acinesia anterior, hipocinesia apical, acinesia septal, IM da parede anterior no ECG de admissão, e acinesia apical. Por fim, o bloqueio de ramo direito está associado tanto com uma grande área de infarto como uma lesão na ADAE.^26-29^

Uma análise da farmacoterapia na alta hospitalar revelou que os pacientes com IC não recebiam o tratamento adequado, particularmente quanto ao uso de antagonista de receptor de mineralocorticoide, uma classe de medicamentos que melhora o remodelamento cardíaco e os desfechos cardiovasculares. Somente 11,0% dos pacientes com IC, dos quais 42,7% foram diagnosticados com FEr, receberam uma prescrição de espironolactona, gerando preocupações sobre a subprescrição de terapia baseada em evidência e um desvio das recomendações de diretrizes.^30^ Vale mencionar que a espironolactona foi o único antagonista da aldosterona aprovado no Brasil até 2021. Apesar da escassa literatura sobre o tema, um estudo recente sugere que preocupações acerca de hipercalemia, hipotensão e ginecomastia são plausíveis para a subprescrição de antagonistas de receptor de mineralocorticoide.^31^ É importante destacar também que os dados usados neste estudo antecedem a recomendação do uso de inibidores de SGLT2 em paciente com IC.^32^

No contexto de acesso limitado à ICPP, como em países de baixa e média renda, novas características, particularmente o papel da trombólise ineficaz, assumem um papel preditivo chave. A alta incidência de IC após o IAMCSST na população estudada (39,9%), principalmente entre os indivíduos em que a trombólise não foi efetiva (47,0%), destaca a necessidade de ICP de resgate apesar da abordagem fármaco-invasiva, uma vez que 29,8% dos pacientes ainda dependeram dessa estratégia para sobreviver após o IAMCSST. Em países de alta renda, como os Estados Unidos, a Suécia, a Dinamarca e a Austrália, a incidência de IC secundária a IM diminuiu drasticamente de 20-46% na era trombolítica para 4-28% na era ICP.^5-10^ Por outro lado, países de renda baixa e média ainda enfrentam desafios pela disponibilidade limitada de ICPPs. Nossos resultados enfatizam a necessidade crítica de se expandir a realização de ICP nos sistemas de saúde de países de renda baixa e média como o Brasil. Ainda, atrasos no tempo entre o início do sintoma à chegada ao hospital e entre a chegada ao hospital e a realização de trombólise também contribuem para a adesão subótima às diretrizes.^33^ A melhoria das redes de assistência pré-hospitalar e a redução desses atrasos poderiam aumentar a probabilidade e trombólise bem-sucedida.^34^

A importância de nosso estudo pode ir além do Brasil, ressoando em outros países de baixa e de média rendas, em que 75% das mortes cardiovasculares ocorrem desproporcionalmente.^35^ Os preditores de IC pós-IAMCSST identificados, no contexto de acesso limitado à ICP, poderiam ser usados como um formato inicial, potencialmente aplicável para estratégias em desenvolvimento em vários países de renda baixa e média que encaram os mesmos desafios. Este estudo também destaca a necessidade de se expandir a disponibilidade de ICPP e de se aplicar as recomendações baseadas em evidências de diretrizes farmacoterapêuticas nesses países. Como uma referência para melhorar o manejo do IAMCSST, nossos resultados fornecem importantes informações para o desenvolvimento de intervenções estratégias e políticas de saúde, abordando a alta carga das doenças cardiovasculares nos países de renda baixa e média.

### Pontos fortes e limitações do estudo

O principal ponto forte deste estudo é o fato de que ele apresenta dados recentes, entre janeiro de 2015 e fevereiro de 2020. Além disso, todos os participantes foram submetidos à angiografia coronária dentro de 24 horas após a trombólise, fornecendo uma perspectiva contemporânea apesar dos recursos limitados dos países de renda baixa e média. Ainda, nosso estudo teve como foco somente pacientes com IAMCSST, o que é um diferencial em relação a outros estudos.^1,5-10,12,17,18,20,21^ Finalmente, o tamanho amostral de 2622 participantes, obtido de uma coorte regionalmente representativo, reforça o poder estatístico e a validade interna.

Uma limitação essencial deste estudo foi seu delineamento retrospectivo. Para abordar o viés de seleção, nós incluímos sequencialmente todos os pacientes admitidos por IAMCSST aos centros participantes durante o período do estudo. Para minimizar problemas de acurácia dos dados e de dados incompletos, utilizamos um formulário padronizado e objetivo para coletar os dados, e um comitê de avaliação revisou os dados. Finalmente, a identificação dos fatores de risco não pode estabelecer causalidade, limitando potenciais intervenções visando reduzir a incidência de IC após IAMCSST.

## Conclusões

Nosso estudo, com base em uma coorte regionalmente representativa de um típico país de renda baixa/média, com acesso limitado à ICP, revelou que homens mais velhos com fatores de risco cardiovascular-renal-metabólico aumentados apresentam maior vulnerabilidade para IC após IAMCSST. A observação de uma alta incidência de IC, particularmente entre os indivíduos em que a trombólise não foi efetiva, destaca a necessidade de se expandir a disponibilidade da ICPP em regiões em que a trombólise primária continua sendo o tratamento padrão. Nossos achados também enfatizam a necessidade de otimizar a farmacoterapia da IC, uma vez que essa medida melhoraria significativamente o desfecho clínico dessa população, principalmente em locais com recursos limitados.

Os insights obtidos neste estudo pioneiro sobre preditores de IC após IAMCSST em um país de renda baixa/média, com acesso limitado à ICP, podem ter implicações em pesquisas, prática clínica e políticas de saúde futuras. Os fatores de risco identificados, incluindo componentes cardiovasculares, renais e metabólicos, e trombólise inefetiva, chamam a atenção para a necessidade de mais estudos a fim de aprimorar os modelos de predição de risco adequados a locais de recursos limitados. Este estudo também destaca o tratamento inadequado de pacientes com IC na alta hospitalar após IAMCSST em países de renda baixa/média, e a necessidade de iniciativas educacionais direcionadas, para melhorar a adesão a diretrizes baseadas em evidências. Intervenções políticas visando expandir a disponibilidade de ICPP em países de renda baixa e média, como enfatizado em nossos resultados, poderiam reduzir significativamente a incidência de IC após IAMCSST. Esses esforços contribuirão para melhorar o tratamento do IAMCSST bem como os desfechos do paciente nesses ambientes de recursos limitados.
