# Supplementary material for: Preditores Clínicos de Insuficiência Cardíaca após IAMCSST: Dados de um País de Renda Média com Acesso Limitado à Intervenção Coronária Percutânea
Source: Arq Bras Cardiol. 2025 Mar 27;122(3):e20240447. [Article in Portuguese] doi: 10.36660/abc.20240447 (PMC12058137; doi:10.36660/abc.20240447)
Supplement: Translation

# Translation: Clinical Predictors of Heart Failure after STEMI: Data from a Middle-Income Country with Limited Access to Percutaneous Coronary Intervention

**DOI:** 10.36660/abc.20240447i

**Authors:** Vinícius C. Fiusa, Andrea D. Stephanus, Victor F. Couto, Gustavo A. Alexim, Thaiene M. M. Severino, Ana Claudia C. Nogueira, Adriana J. B. A. Guimarães, Alexandre Anderson S. M. Soares, Elizabeth Bilevicius, Vivian Batista, Alessandra Staffico, Andrei C. Sposito, Luiz Sérgio F. de Carvalho

**Affiliations:** 1 Brazil Universidade Católica de Brasília, Brasília, DF – Brazil; 2 Brazil Escola Superior de Ciências da Saúde, Brasília, DF – Brazil; 3 Brazil Instituto Aramari Apo, Brasília, DF – Brazil; 4 Brazil Viatris Brasil, São Paulo, SP – Brazil; 5 USA Creighton University - Pharmacy Sciences, Omaha, Nebraska – USA; 6 Brazil Universidade Estadual de Campinas,Campinas, SP – Brazil; 7 Brazil Clarity Healthcare Intelligence, Jundiaí, SP – Brazil

**Keywords:** Heart Failure, ST Elevation Myocardial Infarction, Risk Factors, Pharmacological Thrombolysis

## Abstract

**Background:**

Heart failure (HF) is a common complication of ST-elevation myocardial infarction (STEMI) in low- and middle-income countries (LMICs), where cardiovascular mortality is disproportionately high. Primary percutaneous coronary intervention (PCI) has reduced post-STEMI HF incidence in high-income countries. However, access to this standard of care is poor in LMICs, and data in these settings remain scarce

**Objective:**

To identify predictors of HF following STEMI in a LMIC with limited access to PCI, aiming at better management and outcomes.

**Methods:**

This retrospective cohort study analyzed 2,467 STEMI patients admitted to two Brazilian public hospitals between January/2015 and February/2020. All participants received pharmacological thrombolysis and underwent coronarography within 48h post-admission. The primary outcome was symptomatic HF, defined as dyspnea with chest X-ray evidence of congestion, from 48h post-admission until discharge. Stepwise binary logistic regression was used to identify HF predictors. Significance was defined as p-values<0.05.

**Results:**

The population was 61.9% male, mean age was 58.3±12.6 years, and 39.9% developed post-STEMI HF. HF was more common among older men with cardiovascular-kidney-metabolic (CKM) disease, larger infarcts, and left anterior descending artery involvement. Medications were often underprescribed at discharge, especially aldosterone antagonists (11.0%). HF was notably more frequent among individuals with failed thrombolysis (47.0%).

**Conclusions:**

This regionally representative cohort from a LMIC with limited access to PCI showed that older men with CKM disease are particularly vulnerable to post-STEMI HF, and that HF pharmacotherapy at discharge needs optimization. The high HF incidence among patients with failed thrombolysis highlights the need to expand PCI availability.

## Introduction

ST-elevation myocardial infarction (STEMI) continues to be a major contributor to morbidity and mortality worldwide.^1,2^ STEMI often leads to heart failure (HF), with high healthcare costs and a major impact on patient quality of life and productivity.^3,4^

In recent decades, advances in acute cardiovascular care and revascularization techniques, particularly primary percutaneous coronary intervention (PCI), have led to improved outcomes in high-income countries, such as United States, Sweden, Denmark, and Australia.^5-10^However, this gold-standard strategy is often unavailable in low- and middle-income countries (LMICs), and patients in these settings primarily receive pharmacological thrombolysis, or even no reperfusion therapy.

Recent articles have explored HF risk factors and/or STEMI outcomes after the advent of primary PCI.^11-13^ Studies from the thrombolytic era also investigated those topics in high-income countries.^2^However, to our knowledge, there is no contemporary literature on HF after STEMI in LMICs with limited or no access to PCI.

Therefore, this large multicenter study aims to identify and quantify predictors of HF post-STEMI in a typical LMIC with limited access to PCI. Identifying these predictors paves the way for more effective management strategies and improved outcomes in these resource-limited environments.

## Methods

### Study design, population, and ethics

This study used data from the Brasilia Cardiovascular Registry for Quality of Care and Outcomes (B-CaRe:QCO), a retrospective database of 6,341 patients admitted for acute coronary syndrome to two public tertiary hospitals of Brazil’s Federal District, between January/2011 and February/2020.^14,15^ We included only the subset of patients hospitalized for STEMI, and only those admitted after January/2015, as data on incident HF were not available for patients hospitalized beforehand (n=2,722). We also excluded 275 individuals with HF at baseline and patients with Killip class II-IV at hospital admission. The final cohort encompassed 2,467 eligible patients, as outlined in the STROBE flowchart (Figure 1).

**Figure 1:** – STROBE flowchart. STEMI: ST-elevation myocardial infarction. HF: heart failure.

All participants received pharmacological thrombolysis and underwent coronarography within 48h of admission, at either Hospital de Base do Distrito Federal (HBDF), or at Instituto de Cardiologia e Transplantes do Distrito Federal (ICTDF), both in Brasilia, Brazil. Between 2015 and 2019, these two institutions performed nearly 99% of all coronary angiographies in patients with STEMI in the public health system of Brazil’s Federal District.

Research methodology followed the World Medical Association (WMA)’s Declaration of Helsinki, and was approved by the Institutional Review Board (IRB) of the Instituto de Gestão Estratégica de Saúde do Distrito Federal (IGESDF), which also granted a waiver for informed consent given the de-identified data collection (approval number 28530919.0.1001.8153).

### Data collection and primary outcome

Data were collected through analysis of digital medical record using standardized collection forms. Data categories included: demographics, medical history, index STEMI characteristics, laboratory results, treatment details, and in-hospital outcomes. Laboratory testing followed standard clinical procedures. Coronary anatomy severity, atherosclerotic disease extent, angiographic treatments, TIMI flow grade, and myocardial blush grade (MBG) were determined by analysis of medical reports.

The primary outcome was in-hospital incident HF, defined as dyspnea with chest X-ray evidence of congestion, from 48h post-admission until discharge.

### Statistical analysis

Population characteristics were compared between groups with or without HF. Categorical variables were represented as counts and percentages, and were compared using Pearson’s chi-squared test (or Fisher’s exact test for variables with less than 10 occurrences). Continuous variables were expressed as mean and standard deviation, and were compared using independent Student’s t-test.

Normality was tested using Kolmogorov-Smirnov test and confirmed with graphical inspection. Variables were also tested for absence of multicollinearity. Stepwise binary logistic regression (forward likelihood ratio method) was used to identify and quantify predictors of symptomatic in-hospital post-STEMI HF. The associated odds ratio (OR) and its respective 95% confidence interval (95%CI) were calculated for each predictor identified. The Hosmer-Lemeshow test was used to check if the model is a good fit for the data.

Statistical significance was defined as *p*-values<0.05. Microsoft Excel 2021 for Windows was used for data management. Statistical analyses were conducted on IBM SPSS 26 for Windows.

## Results

From January/2015 to February/2020, 2,467 individuals were admitted for STEMI in two public tertiary hospitals in Brazil’s Federal District. Mean age of the population was 58.3±12.6 years, and most patients were male (61.9%, n=1,520 individuals). Post-STEMI HF was observed in 984 patients (39.9%).

### Baseline population characteristics

HF was more frequent among males and older patients (Table 1). Notably, the odds of developing the condition were significantly lower for those under 60 years (OR: 0.581, 95%CI: 0.491-0.687) compared to those aged 60-79 years (OR: 1.500 (95%CI: 1.262-1.783)) and 80 years and above (OR: 2.241 (95%CI: 1.541-3.259)). Obesity was more prevalent in the HF group, as was hypertension, diabetes, hypothyroidism, chronic kidney disease (CKD), previous coronary arterial disease (CAD), previous myocardial infarction (MI), and previous PCI.

**Table 1:** – Baseline characteristics

Variable	Incident HF post-STEMI		p-value	OR(95%CI)
	No(n=1,483)	Yes(n=984)		
**Demographics**				
Male sex, n (%)	885(59.6)	635(64.5)	0.015*	1.229(1.040-1.452)
Age (years), μ±SD	57.5±13.3	59.5±11.4	<0.001†	-
Age <60 years, n (%)	1,026(69.2)	557(56.6)	<0.001*	0.581(0.491-0.687)
Age 60-79 years, n (%)	408(27.5)	357(36.3)	<0.001*	1.500(1.262-1.783)
Age ≥80 years, n (%)	49(3.3)	70(7.1%)	<0.001*	2.241(1.541-3.259)
BMI (kg/m^2^), μ±SD	26.73±4.50	27.13±4.57	0.032†	-
Underweight (<18.5kg/m^2^), n (%)	22(1.5)	7(0.7)	0.088‡	0.475(0.202-1.115)
Normal Weight (18.5-24.9kg/m^2^), n (%)	559(37.8)	347(35.3)	0.204*	0.897(0.758-1.061)
Overweight (25-29.9kg/m^2^), n (%)	613(41.5)	403(41.0)	0.814*	0.980(0.832-1.155)
Obese I (30-34.9kg/m^2^), n (%)	219(14.8)	166(16.9)	0.166*	1.168(0.937-1.456)
Obese II (35-39.9kg/m^2^), n (%)	45(3.0)	46(4.7)	0.035*	1.563(1.028-2.377)
Obese III (≥40kg/m^2^), n (%)	20(1.4)	14(1.4)	0.882*	1.053(0.529-2.095)
**Medical history**				
Obesity, n (%)	260(17.5)	219(22.3)	0.004*	1.347(1.101-1.647)
Hypertension, n (%)	863(58.2)	614(62.4)	0.037*	1.192(1.011-1.406)
Diabetes, n (%)	387(26.1)	349(35.5)	<0.001*	1.557(1.307-1.853)
Dyslipidemia, n (%)	709(47.8)	507(51.5)	0.071*	1.160(0.988-1.363)
Smoking history, n (%)	960(64.7)	610(62.0)	0.166*	0.889(0.752-1.050)
Alcohol abuse history, n (%)	183(12.3)	118(12.0)	0.796*	0.968(0.756-1.239)
Illicit drugs abuse history, n(%)	64(4.3)	31(3.2)	0.141*	0.721(0.466-1.116)
CVD family history, n (%)	319(21.5)	197(20.0)	0.373*	0.913(0.748-1.115)
Hypothyroidism, n (%)	73(4.9)	72(7.3)	0.013*	1.525(1.090-2.134)
**End-organ damage**				
CKD, n (%)	68(4.6)	90(9.1)	<0.001*	2.095(1.512-2.902)
Prior CAD, (%)	399(26.9)	324(32.9)	0.001*	1.334(1.119-1.590)
Prior angina, n (%)	330(22.3)	243(24.7)	0.159*	1.146(0.948-1.385)
Prior MI, n (%)	105(7.1)	128(13.0)	<0.001*	1.962(1.495-2.575)
Prior PCI, n (%)	47(3.2)	70(7.1)	<0.001*	2.340(1.602-3.418)
Prior CABG, n (%)	26(1.8)	17(1.7)	0.962*	0.985(0.532-1.825)
Prior stroke, n (%)	51(3.4)	44(4.5)	0.192*	1.314(0.871-1.984)
Prior PAD, n (%)	57(3.8)	51(5.2)	0.111*	1.368(0.929-2.013)

*Pearson’s chi-squared test. †Independent Student’s t-test; ‡Fisher’s exact test; bolded p-values indicate statistical significance at a threshold of p<0.05; OR: odds ratio; CI: confidence interval; n: number of individuals; μ: mean value; SD: standard deviation; HF: heart failure; STEMI: ST-elevation myocardial infarction; BMI: body mass index; CVD: cardiovascular disease; CKD: chronic kidney disease; CAD: coronary arterial disease; MI: myocardial infarction; PCI: percutaneous coronary intervention; CABG: coronary artery bypass grafting; PAD: peripheral artery disease.

### Characteristics of index STEMI

Regarding symptomatology at admission, patients who developed HF presented more often with syncope and cardiac arrest (Table 2). They also had lower systolic and diastolic blood pressures, as well as higher GRACE and CRUSADE scores. Furthermore, HF patients more often had anterior myocardial infarction and right bundle branch block (RBBB) on admission electrocardiogram (ECG). Pathological Q-waves were more common in leads V1-V4 (anterior), V1-V6 (anterolateral), and V4R (right), and less common in leads V5-V6 (lateral) and DII-DIII/AVF (inferior).

**Table 2:** – Characteristics of index ST-elevation myocardial infarction (STEMI)

Variable	Incident HF post-STEMI		p-value	OR(95%CI)
	No(n=1,483)	Yes(n=984)		
**Symptomatology at admission**				
Syncope, n (%)	12(0.8)	20(2.0)	0.009*	2.543(1.238-5.226)
Cardiac arrest, n (%)	4(0.3)	22(2.2)	<0.001‡	8.450(2.903-24.597)
Systolic BP (mmHg), μ±SD	136.3±25.1	129.7±30.0	<0.001†	-
Diastolic BP (mmHg), μ±SD	83.1±16.3	81.1±20.5	<0.001†	-
**Risk stratification at admission**				
GRACE score, μ±SD	99.2±24.5	141.9±43.1	<0.001†	-
CRUSADE score, μ±SD	21.6±12.2	33.7±15.0	<0.001†	-
**Admission ECG**				
Anterior wall MI, n (%)	495(33.4)	628(63.8)	<0.001*	3.521(2.974-4.169)
Right bundle branch block, n (%)	6(0.4)	16(1.6)	0.002‡	4.069(1.587-10.435)
Left bundle branch block, n (%)	4(0.3)	6(0.6)	0.211‡	2.268(0.638-8.059)
2nd/3rd-degree AV block, n (%)	5(0.3)	7(0.7)	0.240‡	2.118(0.670-6.692)
Septal (V1-V3) path. Q-waves, n (%)	80(5.4)	55(5.6)	0.835*	1.038(0.729-1.478)
Anterior (V1-V4) path. Q-waves, n (%)	191(12.9)	191(19.4)	<0.001*	1.629(1.309-2.029)
Anterolateral (V1-V6) path. Q-waves, n (%)	225(15.2)	383(38.9)	<0.001*	3.563(2.943-4.314)
Lateral (V5-V6) path. Q-waves, n (%)	80(5.4)	30(3.0)	0.006*	0.551(0.360-0.846)
Right (V4R) path. Q-waves, n (%)	129(8.7)	110(11.2)	0.041*	1.321(1.010-1.727)
Inferior (DI, DII, AVF) path. Q-waves, n (%)	649(43.8)	211(21.4)	<0.001*	0.351(0.292-0.421)
**Laboratory results**				
Admission hs-cTnI (ng/L), μ±SD	5,042±5,978	8,436±9,071	<0.001†	-
Peak hs-cTnI (ng/L), μ±SD	6,164±5,658	11,145±10,992	<0.001†	-
AST (U/L), μ±SD	167±139	267±198	<0.001†	-
ALT (U/L), μ±SD	49.4±48.5	95.7±185.7	<0.001†	-
Creatinine (mg/dL), μ±SD	0.92±0.40	1.11±0.87	<0.001†	-
GFR (mL/min), μ±SD	96.5±34.3	85.4±35.3	<0.001†	-
Blood glucose (mg/dL), μ±SD	132.6±56.5	166.0±85.6	<0.001†	-
HbA1c (%), μ±SD	6.55±1.81	6.95±2.23	<0.001†	-
TSH (mIU/L), μ±SD	2.16±3.99	2.81±6.21	0.007†	-
FT4 (ng/dL), μ±SD	1.29±0.63	1.30±0.62	0.744†	-
Total cholesterol (mg/dL), μ±SD	201.49±45.5	198.2±48.5	0.111†	-
HDL-C (mg/dL), μ±SD	41.5±12.3	41.3±12.5	0.637†	-
LDL-C (mg/dL), μ±SD	130.0±39.5	125.2±40.9	0.010†	-
Triglycerides (mg/dL), μ±SD	158.2±109.7	158.9±119.2	0.876†	-
Hemoglobin (g/dL), μ±SD	14.55±1.70	14.50±1.96	0.053†	-
Hematocrit (%), μ±SD	43.03±4.84	42.86±5.72	0.448†	-
PT (s), μ±SD	85.3±12.2	81.4±15.4	<0.001†	-
PTT (s), μ±SD	34.9±13.0	38.0±20.2	<0.001†	-

*Pearson’s chi-squared test. †Independent Student’s t-test. ‡Fisher’s exact test. Bolded p-values indicate statistical significance at a threshold of p<0.05. OR: odds ratio. CI: confidence interval. n: number of individuals. μ: mean value. SD: standard deviation. STEMI: ST-elevation myocardial infarction. HF: heart failure. BP: blood pressure. AV: atrioventricular; hs-cTnI: high-sensitivity cardiac troponin I. AST: aspartate aminotransferase. ALT: alanine aminotransferase. GFR: glomerular filtration rate. HbA1c: hemoglobin A1c. TSH: thyroid-stimulating hormone. FT4: free thyroxine. HDL-C: high density lipoprotein cholesterol. LDL-C: low density lipoprotein cholesterol. PT: prothrombin time. PTT: partial thromboplastin time.

High-sensitivity cardiac troponin I (hs-cTnI) at admission was higher in the HF group, as was its peak levels and aspartate aminotransferase (AST), indicating more extensive myocardial injury. Renal impairment was also more common in the HF group, with higher creatinine and lower glomerular filtration rate (GFR). HF patients also had elevated blood glucose and hemoglobin A1c (HbA1c), and lower low-density lipoprotein cholesterol (LDL-C). Also, shorter prothrombin time (PT) and prolonged partial thromboplastin time (PTT) were seen among those with HF.

### Treatment details

Pharmaco-invasive treatment strategy was strongly and inversely associated with incident post-STEMI HF (OR: 0.280, 95%CI: 0.235-0.334) (Table 3). In contrast, individuals who underwent rescue PCI after failing to meet reperfusion criteria more often developed HF (OR: 3.920, 95%CI: 3.269-4.699). Shorter times from symptom onset to hospital presentation and from thrombolysis to catheterization were seen in the HF group, likely reflecting the logistical prioritization of more severe presentations given limited capacity of our public healthcare system.

**Table 3:** – Treatment details

Variable	Incident HF post-STEMI		p-value	OR(95%CI)
	No(n=1,483)	Yes(n=984)		
**Treatment details**				
Pharmaco-invasive, n (%)	1,164(78.5)	498(50.6)	<0.001*	0.280(0.235-0.334)
Rescue PCI, n (%)	273(18.4)	462(47.0)	<0.001*	3.920(3.269-4.699)
2nd cath., n (%)	110(7.5)	91(9.3)	0.106*	1.270(0.950-1.698)
Angioplasty on the 2^nd^ cath., n (%)	81(7.6)	61(8.1)	0.669*	1.078(0.763-1.524)
Onset-primary hospital (min), μ±SD	158±135	146±126	0.036†	-
Door-needle (min), μ±SD	181±3,436	98±92	0.449†	-
Thrombolysis-cath. (min), μ±SD	1,392±1,220	1,059±1,186	<0.001†	-
**Catheterization and PCI details**				
Use of GPIIb/IIIa inhibitor, n (%)	50(3.4)	66(6.7)	<0.001*	2.061(1.414-3.003)
Use of nitroprusside, n (%)	5(0.3)	10(1.0)	0.034‡	3.035(1.034-8.906)
Use of adenosine, n (%)	38(2.6)	61(6.2)	<0.001*	2.513(1.662-3.800)
No. of conventional stents, μ±SD	0.83±0.73	0.93±0.75	0.001†	-
No. of drug-eluting stents, μ±SD	0.09±0.35	0.07±0.34	0.136†	-
TIMI-3 flow post-PCI, n (%)	1,058(83.4)	601(69.0)	<0.001*	0.444(0.361-0.545)
MBG grade 3 post-PCI, n (%)	845(69.7)	393(49.0)	<0.001*	0.418(0.348-0.503)
Cath. duration (min), μ±SD	90±1,181	62±29	0.460†	-
**Echocardiography 48-72h after admission**				
LVEF (%), μ±SD	54.9±7.4	41.0±9.8	<0.001†	-
LVEF <40%, n (%)	1(0.1)	367(42.7)	<0.001‡	851.8(119.3-6,080.8)
Hypokinesia anterior, n (%)	192(12.9)	254(25.8)	<0.001*	2.340(1.900-2.881)
Hypokinesia septal, n (%)	373(25.2)	257(26.1)	0.590*	1.052(0.875-1.265)
Hypokinesia lateral, n (%)	125(8.4)	160(16.3)	<0.001*	2.110(1.644-2.707)
Hypokinesia right, n (%)	3(0.2)	15(1.5)	<0.001‡	7.637(2.205-26.449)
Hypokinesia inferior, n (%)	396(26.7)	211(21.4)	0.003*	0.749(0.619-0.907)
Hypokinesia apical, n (%)	77(5.2)	91(9.2)	<0.001*	1.861(1.358-2.549)
Hypokinesia dorsal, n (%)	273(18.4)	130(13.2)	0.001*	0.675(0.538-0.846)
Akinesia anterior, n (%)	124(8.4)	373(37.9)	<0.001*	6.691(5.345-8.374)
Akinesia septal, n (%)	251(16.9)	467(47.5)	<0.001*	4.434(3.686-5.333)
Akinesia lateral, n (%)	39(2.6)	90(8.1)	<0.001*	3.277(2.215-4.848)
Akinesia right, n (%)	0(0.0)	2(0.2)	0.159‡	0.398(0.380-0.418)
Akinesia inferior, n (%)	382(25.8)	264(26.8)	0.554*	1.057(0.880-1.269)
Akinesia apical, n (%)	188(12.7)	431(43.8)	<0.001*	5.369(4.404-6.545)
Akinesia dorsal, n (%)	108(7.3)	105(10.7)	0.003*	1.521(1.148-2.016)
**Pharmacotherapy at discharge (or death)**				
ACE inhibitors, n (%)	661(44.6)	413(41.9)	0.202*	0.899(0.764-1.058)
Angiotensin receptor blockers, n (%)	351(23.7)	167(16.9)	<0.001*	0.659(0.537-0.809)
Beta-blockers, n (%)	1,003(67.6)	656(66.6)	0.616*	0.957(0.806-1.136)
Aldosterone antagonists, n (%)	64(4.3)	109(11.0)	<0.001*	2.762(2.005-3.803)
Furosemide, n (%)	86(5.8)	110(11.1)	<0.001*	2.044(1.522-2.744)
Calcium channel blockers, n (%)	320(21.6)	145(14.7)	<0.001*	0.628(0.506-0.779)
Statins, n(%)	1,226(82.7)	815(82.8)	0.920*	1.010(0.816-1.251)
Acetylsalicylic acid, n (%)	1,449(97.7)	965(98.8)	0.543*	1.191(0.675-2.101)
Clopidogrel, n (%)	1,035(69.8)	667(67.7)	0.291*	0.910(0.765-1.083)
Prasugrel, n (%)	214(14.4)	260(26.4)	<0.001*	2.129(1.738-2.607)
Ticagrelor, n (%)	33(2.2)	35(3.5)	0.047*	1.620(1.000-2.625)
Anticoagulants, n (%)	46(3.1)	56(5.6)	0.001*	1.885(1.265-2.808)

*Pearson’s chi-squared test. †Independent Student’s t-test. ‡Fisher’s exact test. Bolded p-values indicate statistical significance at a threshold of p<0.05. OR: odds ratio. CI: confidence interval. n: number of individuals. μ: mean value. SD: standard deviation. STEMI: ST-elevation myocardial infarction. HF: heart failure. PCI: percutaneous coronary intervention. LVEF: left ventricular ejection fraction. ACE: angiotensin-converting-enzyme; MBG: myocardial blush grade.

Regarding PCI details, incident HF was more common among those who received GPIIb/IIIa inhibitors and nitroprusside during catheterization, and those with more conventional stents implanted. Final TIMI-3 flow and myocardial blush grade 3 were less frequently achieved in the HF group.

HF patients had lower mean left ventricular ejection fraction (LVEF) and more frequent reduced ejection fraction (rEF) (LVEF <40%) on echocardiogram at 48-72h of admission. Motility changes in the HF group were mainly associated with left anterior descending artery (LAD) involvement.

At hospital discharge (or death), HF patients were less prescribed angiotensin receptor blockers (ARBs) and calcium channel blockers. On the other hand, this group more frequently received aldosterone antagonists, furosemide, prasugrel, ticagrelor, and anticoagulants.

### In-hospital outcomes

Recurrent angina and MI occurred more often in the HF group (Table 4). Other cardiovascular complications were also associated with incident HF, including: atrial fibrillation, total atrioventricular block, mitral regurgitation, cardiac arrest, ischemic and hemorrhagic stroke, minor and major bleeding, and blood transfusion. Mean hospitalization stay was longer in the HF group, along with higher all-cause in-hospital mortality.

**Table 4:** – In-hospital outcomes

Variable	Incident HF post-STEMI		p-value	OR(95%CI)
	No(n=1,483)	Yes(n=984)		
Recurrent angina, n (%)	32(2.2)	35(3.6)	0.036*	1.672(1.028-2.720)
Recurrent MI, n (%)	12(0.8)	28(2.8)	<0.001*	3.590(1.817-7.095)
Atrial fibrillation, n (%)	31(2.1)	55(5.6)	<0.001*	2.773(1.772-4.339)
3rd degree atrioventricular block, n (%)	48(3.2)	70(7.1)	<0.001*	2.290(1.571-3.337)
Mitral regurgitation, n (%)	3(0.2)	9(0.9)	0.017‡	4.554(1.230-16.863)
Cardiac arrest, n (%)	5(0.3)	24(2.4)	<0.001‡	7.385(2.808-19.422)
Ischemic stroke, n (%)	6(0.4)	21(2.1)	<0.001‡	5.368(2.159-13.348)
Hemorrhagic stroke, n (%)	3(0.2)	13(1.3)	0.001‡	6.605(1.877-23.238)
Minor bleeding, n (%)	28(1.9)	38(3.9)	0.003*	2.087(1.272-3.424)
Major bleeding, n (%)	11(0.7)	51(5.2)	<0.001*	7.315(3.793-14.106)
Blood transfusion, n (%)	7(0.5)	31(3.2)	<0.001‡	6.859(3.008-15.639)
Hospitalization length (days), μ±SD	4.01±3.15	6.59±7.36	<0.001†	-
All-cause in-hospital death, n (%)	12(0.8)	110(11.2)	<0.001*	15.428(8.453-28.160)

*Pearson’s chi-squared test. †Independent Student’s t-test. ‡Fisher’s exact test. Bolded p-values indicate statistical significance at a threshold of p<0.05. OR: odds ratio. CI: confidence interval. n: number of individuals. μ: mean value. SD: standard deviation. STEMI: ST-elevation myocardial infarction. HF: heart failure. MI: myocardial infarction.

### Predicting in-hospital HF post-STEMI

We conducted a stepwise binary logistic regression (forward likelihood ratio method) to identify and quantify significant predictors of incident in-hospital HF following STEMI (Table 5). This analysis resulted in a statistically significant model [Nagelkerke R^2^=0.550, p=0.039] that correctly classified 82.3% of cases. The Hosmer-Lemeshow test indicated a good model fit (p=0.746).

**Table 5:** – Predictors of incident in-hospital HF post-STEMI identified by stepwise binary logistic regression (forward likelihood ratio method)

Variable	B	SE	Wald	p-value	OR(95%CI)
Risk predictors for in-hospital HF post-STEMI					
AST (increments of 100U/L)	0.373	0.061	37.398	<0.001*	1.453(1.289-1.637)
Right (V4R) path. Q-waves (on admission ECG)	1.770	0.318	30.920	<0.001*	5.868(3.145-10.950)
Anterior akinesia (48-72h after admission)	1.159	0.304	14.549	<0.001*	3.187(1.757-5.782)
Rescue PCI	0.787	0.215	13.400	<0.001*	2.197(1.441-3.348)
Apical hypokinesia (48-72h after admission)	1.260	0.349	13.005	<0.001*	3.524(1.777-6.987)
Septal akinesia (48-72h after admission)	0.869	0.249	12.207	<0.001*	2.384(1.464-3.880)
Cath. duration (h)	0.750	0.216	12.052	0.001*	2.117(1.386-3.233)
Hypothyroidism	1.114	0.361	9.547	0.002*	3.047(1.503-6.177)
Prior MI	0.915	0.307	8.875	0.003*	2.497(1.368-4.559)
Dorsal akinesia (48-72h after admission)	0.940	0.329	8.157	0.004*	2.560(1.343-4.880)
Blood glucose (increments of 100mg/dL)	0.371	0.147	6.351	0.012*	1.449(1.086-1.933)
Right bundle branch block (on admission ECG)	2.442	1.022	5.712	0.017*	11.501(1.552-85.245)
Lateral hypokinesia (48h after admission)	0.705	0.297	5.621	0.018*	2.023(1.130-3.622)
Creatinine (mg/dL)	0.566	0.249	5.168	0.023*	1.761(1.081-2.870)
Smoking history	0.478	0.211	5.142	0.023*	1.613(1.067-2.438)
Obesity	0.492	0.236	4.343	0.037*	1.636(1.030-2.600)
Anterior wall MI (on admission ECG)	0.553	0.267	4.286	0.038*	1.739(1.030-2.936)
Apical akinesia (48-72h after admission)	0.544	0.286	3.609	0.057*	1.722(0.983-3.017)
Protective predictors for in-hospital post-STEMI HF					
SBP (mmHg) (at admission)	-0.017	0.004	21.893	<0.001*	0.983(0.976-0.990)
Age <60 years	-0.868	0.204	18.131	<0.001*	0.420(0.282-0.626)
TIMI-3 flow post-PCI	-0.880	0.285	9.522	0.002*	0.415(0.237-0.726)
PT (s)	-0.018	0.007	7.161	0.007*	0.982(0.969-0.995)

*Stepwise binary logistic regression (forward likelihood ratio method). Bolded p-values indicate statistical significance at a threshold of p<0.05. SE: standard error. OR: odds ratio. CI: confidence interval. STEMI: ST-elevation myocardial infarction. HF: heart failure. ECG: electrocardiogram. AST: aspartate aminotransferase. PCI: percutaneous coronary intervention. MI: myocardial infarction. hs-cTnI: high-sensitivity cardiac troponin I. SBP: systolic blood pressure. PT: prothrombin time.

Risk predictors of incident post-STEMI HF identified include: AST (in increments of 100U/L), V4R pathological Q-waves, anterior akinesia, rescue PCI, apical hypokinesia, septal akinesia, catheterization duration (in hours), hypothyroidism, prior MI, dorsal akinesia, blood glucose (in increments of 100mg/dL), RBBB, lateral hypokinesia, creatinine (mg/dL), smoking history, obesity, anterior MI, and apical akinesia.

As for protective predictors, the following factors were identified: higher systolic blood pressure (SBP) at admission, age <60 years, TIMI-3 flow after PCI, and PT (in seconds).

## Discussion

To our knowledge, this is a pioneering study in identifying predictors of both risk and protection for HF after STEMI in a LMIC with limited access to primary PCI (Central Illustration). Patients who developed HF showed a clear profile of increased cardiovascular risk, marked by older age, male sex, metabolic dysfunction, and already established end-organ damage. Larger infarcts and LAD involvement were also found to be predictive of HF. Additionally, HF patients were notably underprescribed medications that improve cardiac remodeling, particularly aldosterone antagonists. Lastly, the high incidence of HF, especially among those with failed thrombolysis, underscore the imperative to expand primary PCI feasibility in LMICs like Brazil.

The increased cardiovascular-kidney-metabolic (CKM) risk observed among patients who developed HF after STEMI aligns with previous studies.^13,16-18^Interestingly, almost all cardiovascular (hypertension, prior CAD), kidney (chronic kidney disease), and metabolic (obesity, diabetes, hypothyroidism) components were more prevalent in the HF group. The exception was dyslipidemia, where HF patients exhibited lower LDL-C levels. This result was also observed in other studies and is mostly explained by the acute inflammatory response associated with the infarction.^13,18,19^

Our findings also corroborate the well-established associations of infarct size and anterior MI with post-MI HF.^13,18,20,21^ Upon hospital admission, a lower blood pressure suggests myocardial injury severe enough to cause some degree of hemodynamic impairment. Higher serum levels of hs-cTnI and AST, which correlate with a larger infarcted area, were also observed in the HF group.^22-25^LAD involvement played a significant role in our predictive model, encompassing several components, such as anterior akinesia, apical hypokinesia, septal akinesia, anterior wall MI on admission ECG, and apical akinesia. Lastly, RBBB is associated with both a large infarct area and LAD lesion.^26-29^

An analysis of pharmacotherapy at hospital discharge revealed that HF patients were undertreated, particularly for mineralocorticoid receptor antagonist (MRAs), a class of medication that improve cardiac remodeling and cardiovascular outcomes. Only 11.0% of HF patients, among whom 42.7% were diagnosed with rEF, were prescribed spironolactone, raising concerns about the underprescribing of evidence-based therapy and a deviation from guideline recommendations.^30^ It is worth mentioning that spironolactone was the only aldosterone antagonist approved for use in Brazil up until 2021. Despite the limited literature on this topic, a recent study suggests that concerns regarding hyperkalemia, hypotension and gynecomastia are plausible explanations for the underprescribing of MRAs.^31^It is also important to highlight that the data used in this study precede the recommendation for the use of SGLT2 inhibitors in patients with HF.^32^

In the settings of limited access to primary PCI, as seen in LMICs, new features, particularly the role of failed thrombolysis, take a key predictive role. The high incidence of post-STEMI HF in the studied population (39.9%), especially among individuals with failed thrombolysis (47.0%), highlights the ongoing need for rescue PCI despite the systematic pharmaco-invasive approach, as 29.8% still depended on this strategy to survive after STEMI. In high-income countries, such as United States, Sweden, Denmark, and Australia, the incidence of HF complicating MI has dramatically declined from 20-46% in the thrombolytic era to 4-28% in the PCI era.^5-10^ In contrast, LMICs still face significant challenges with limited primary PCI availability. Our results emphasize the critical need to expand primary PCI capacity in healthcare systems of LMICs like Brazil. Additionally, delays in the time from symptom onset to hospital arrival and from hospital arrival to thrombolysis also contribute to suboptimal adherence to guidelines.^33^ Improving pre-hospital care networks and reducing these delays could enhance the likelihood of successful thrombolysis.^34^

The importance of our study may go beyond Brazil, resonating in LMICs globally, where more than 75% of cardiovascular deaths disproportionally occur.^35^The identified predictors of post-STEMI HF, within the context of limited access to PCI, could be used as a start template, potentially applicable for developing strategies in numerous LMICs facing similar challenges. This study also highlights the need to expand primary PCI availability and to enforce evidence-based pharmacotherapy guidelines in LMICs. As a benchmark for improving STEMI care, our findings provide important information for strategic interventions and healthcare policies, addressing the high burden of cardiovascular diseases across LMICs.

### Strengths and limitations

The main strength of this study is the fact that it provides up-to-date data, from January 2015 to February 2020. Additionally, all participants underwent coronarography within 24h of thrombolysis, providing a contemporary perspective despite the limited resources of a LMIC. Furthermore, our study focused exclusively on STEMI patients, a notable distinction from several previous studies.^1,5-10,12,17,18,20,21^ Lastly, the substantial sample size, of 2,622 participants, drawn from a regionally-representative cohort, bolsters statistical power and internal validity.

A core limitation of this study was its retrospective design. To address selection bias, we sequentially included all patients admitted for STEMI to the participating centers during the study period. To minimize problems arising from data accuracy and completeness, a standardized and objective collection form was used, and an adjudication committee reviewed the data. Lastly, the identification of risk factors cannot establish causality, limiting potential interventions targeted to reduce incident post-STEMI HF.

## Conclusions

Our study, based on a regionally-representative cohort from a typical LMIC with limited access to PCI, revealed that older men with increased CKM risk face greater vulnerability to HF following STEMI. The observed high incidence of HF, particularly among individuals with failed thrombolysis, highlights the need to expand primary PCI availability in regions where primary thrombolysis remains the standard of care. Our findings also emphasize the need to optimize HF pharmacotherapy, as this measure would significantly improve the clinical outcome of this population, especially in resource-limited settings.

The insights gained from our pioneering study on post-STEMI HF predictors in an LMIC with limited PCI access may hold important implications for future research, clinical practice, and health policies. The identified risk factors, including CKM components and failed thrombolysis, call for further investigations to refine risk prediction models tailored to resource-constrained settings. This study also underscores pharmacological undertreatment of HF patients at hospital discharge after STEMI in LMICs, advocating for targeted educational initiatives to improve adherence to evidence-based guidelines. Policy interventions aimed at expanding primary PCI availability in LMICs, as emphasized by our findings, could significantly reduce the incidence of post-STEMI HF. Ultimately, these efforts contribute to enhanced STEMI care and improved patient outcomes in these resource-limited environments.
